# Fabrication of green poly(vinyl alcohol) nanofibers using natural deep eutectic solvent for fast-dissolving drug delivery†

**DOI:** 10.1039/d0ra08755f

**Published:** 2021-01-04

**Authors:** Qingmiao Zhang, Zhuangsheng Lin, Wenkun Zhang, Ting Huang, Jingjing Jiang, Yu Ren, Ruiqi Zhang, Wen Li, Xiaoli Zhang, Qin Tu

**Affiliations:** College of Chemistry & Pharmacy, and Life Science, Northwest A&F University Yangling Shaanxi 712100 P. R. China xiaoliflyhighly@163.com tuqin@nwsuaf.edu.cn +8618702996824 +8618702996824; Department of Chemistry and Chemical Biology, Northeastern University 102 HTT (Hurtig Hall) Boston MA 02115 USA

## Abstract

Fast-dissolving drug delivery systems are essential to drug delivery owing to the enhanced drug solubility, controlled drug concentration, target and rapid drug delivery. In this study, we developed fast-dissolving drug delivery systems using honey and acetylsalicylic acid-embedded poly(vinyl alcohol) (PVA) nanofibers based on natural deep eutectic solvent (DES). The efficacy of our fast-dissolving drug delivery system was tested by incorporating honey and acetylsalicylic acid in the PVA nanofibers. Firstly, the morphology and structure of the functional PVA–DES nanofibers (PVA–DES–honey and PVA–DES–ASA) were observed and analyzed, which proved the successful preparation of functional PVA–DES nanofibers. NIH/3T3 and HepG2 cells incubated on the nanofiber had more than 90% of cell viability, suggesting our materials were biocompatible and non-toxic. The nanofiber materials dissolved rapidly in artificial saliva solutions, suggesting potential use of our materials for fast dissolving drug delivery in oral cavities. The honey incorporated PVA nanofiber (PVA–DES–honey) showed a total bacterial reduction of 37.0% and 37.9% against *E. coli* and *S. aureus*, respectively, after 6 hour incubation in bacterial cultures. Furthermore, *in vivo* study proved that the PVA–DES–honey nanofibers accelerated the wound healing process, and they improved the wound healing rate on rat skin to 85.2% after 6 days of surgery, when compared to the control PVA (68.2%) and PVA–DES (76.3%) nanofibers. Overall, the nanofiber materials reported in our study showed potential as a green and biocompatible fast-dissolving drug delivery system and can be used for pharmaceutical fields, such as antibacterial wound dressing and oral ulcer stickers.

## Abstract 2

We report an environmental friendly method to construct honey/ASA embedded poly(vinyl alcohol) nanofibers based on natural deep eutectic solvent for fast-dissolving drug delivery firstly.

## Graphical abstract

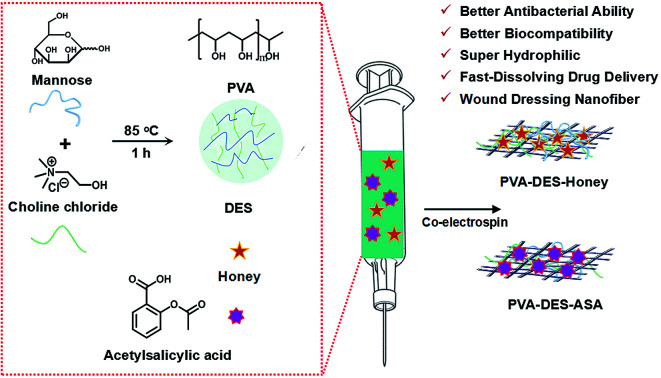

## 1. Introduction

Fast-dissolving drug delivery systems are a promising pathway for target drug delivery within a given range.^1^ They have an attractive prospect in the field of new drug delivery due to advantages, such as enhancing drug solubility, rapid drug release rate, and targeted drug delivery.^2,3^ This technology can enhance the safety and efficacy of the drug by controlling the concentration of the drug, to deliver a target amount of the drug to the target site and release it quickly to maximize the therapeutic effect. Subsequently, side effects caused by changes in drug concentration can be reduced.^3–5^ For example, in the oral cavity, these delivery systems can quickly dissolve when in contact with saliva. The released drugs can be absorbed into the oral cavity, or sucked down into the pharynx and esophagus through the saliva, without water for delivery. By directly releasing the drug in the mouth, it enables rapid onset of action, reduces the side effects of the drug on the gastrointestinal tract, and improves bioavailability and therapeutic potential of the drug.^6,7^

Currently, nanofiber membranes prepared by electrospinning are usually used in wound dressings, biosensing, tissue engineering scaffolds, artificial organs, and drug delivery.^6,8,9^ The nanofibers prepared by electrospinning have several features, such as similar structure to natural extracellular matrix, great porosity, high surface area to volume ratio, good liquid absorption, and 3D continuous web structure. These features of electrospun nanofiber membranes not only promote the rapid wetting of the surface, but also lead to the rapid release of active components when in contact with dissolving media. Electrospun nanofiber membranes can be used to encapsulated biomolecules and have broad prospects for drug delivery applications.^6,10^ Drugs can be embedded into nanofiber membranes in amorphous or nanocrystalline form to produce flexible sheets very similar to the extracellular membrane for drug loading,^2^ with high drug loading capacity and high encapsulation efficiency.^4^ At the same time, the porous structure of nanofibers can creat a suitable moist environment for the wound, which is conducive to wound healing.^6,11^

In this study, we aimed to produce nanofibrous membranes with rapidly dissolve ability by electrospinning, to encapsulate different functional drugs, as a new fast-dissolving drug delivery systems. Therefore, the degradation products cannot be toxic and should be readily reabsorbed, or excreted, by the organism. In addition, to achieve rapid dissolution and drug release, nanofiber materials with appropriate water-solubility are needed.^7^ Polyvinyl alcohol (PVA) is a commercial, inexpensive, water-soluble material approved by the FDA.^12^ Due to the excellent hydrophilicity, biocompatibility, good mechanical properties, and biodegradability, PVA is generally used in various biomedical fields such as pharmaceutical preparations, tissue engineering scaffolds and wound dressings.^13,14^ To develop fast dissolving nanofibers for fast-dissolving drug delivery systems, we used PVA nanofiber as a drug carrier. Generally, polymers need to be dissolved in organic solvents to be spun into nanofibers, but the good water solubility of PVA can make it “green” electrospinning for biomedical applications. This good property can improve the solubility of drugs.

Due to the global awareness of environmental sustainability, there is an increasing demand to develop green technologies in the pharmaceutical field. Natural deep eutectic solvents (DES) is a new green solvent with good solubility, low toxicity, good biocompatibility, reproducibility, and ease of synthesis. At the same time, DES can improve permeability and dissolution the active drug components (API), comparing to pure forms of API, therefore, are generally used in drug delivery systems.^15^ DES was formed by the hydrogen-bond acceptor and donor. In appropriate conditions, the melting point will be greatly reduced compared with the pure component.^16,17^

In this study, we aimed to produce nanofibrous membranes with rapid dissolve ability to encapsulate different functional drugs by electrospinning, and use it as new fast-dissolving drug delivery systems. A biocompatible PVA polymer was dissolved in green DES and was electrospun into nanofiber materials. Choline chloride and mannose were chosen as a hydrogen bond acceptor and donor, respectively to prepare DES with good biocompatibility. Choline chloride is a common hydrogen-bond acceptor to synthesize DES. Choline chloride also is an important nutrient that can be extracted from biomass, with low cost, biodegradability and good biocompatibility. The quaternary ammonium salt component also has excellent antibacterial and anti-inflammatory effects.^18,19^ Mannose is currently the only clinically used sugar nutrient that can regulate the immune system, prevent bacterial infections, heal wounds and fight inflammation.^20^ To test the efficacy of the PVA nanofiber as a fast-dissolving drug delivery system, we used honey and acetylsalicylic acid (aspirin, ASA) as model drugs. The dissolution of nanofiber materials was tested in water and artificial saliva solutions. The cytocompatibility of the materials was tested using cell viability analysis in NIH/3T3 and HepG2 cell cultures. The antimicrobial and wound healing efficacy of the honey incorporated nanofibers were tested in *E. coli* and *S. aureus* bacterial cultures and on rat skins, respectively.

## 2. Experimental

### 2.1 Materials

Polyvinyl alcohol (*M*_w_: 205 000) and mannose were purchased from Aladdin (Shanghai, China). Acetylsalicylic acid and choline chloride were obtained from Adamas-Beta (Shanghai, China). Honey (acacia) was obtained from Shanxi province in China and stored at 4 °C. Unless otherwise stated, all reagents and materials were used directly.

### 2.2 Fabrication of PVA–DES–honey/ASA nanofibers

#### 2.2.1 Preparation of DES

The DES solution was prepared according to previous reports.^7,16,17^ Choline chloride and mannose were mixed on a molar ratio of 2 : 1 and stirred at 80 °C for 1 h to obtain a transparent and homogenous DES solution. After the mixture was cooled to room temperature, it remained liquid.

#### 2.2.2 Preparation of the electrospun solutions

**Scheme 1:**
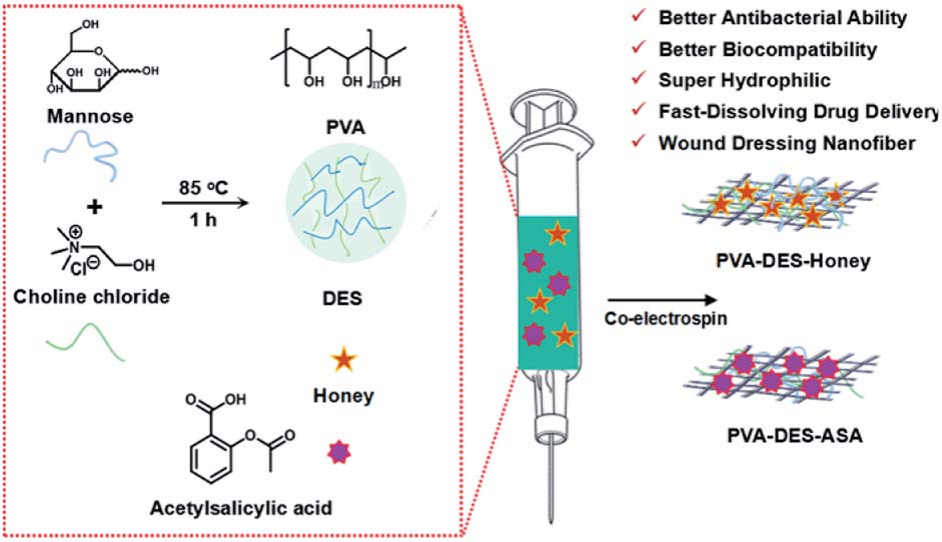
Schematic diagram representation the fabrication of honey and acetylsalicylic acid-embedded poly(vinyl alcohol) (PVA) nanofibers based on natural DES.

The electrospinning solutions of PVA, PVA–DES and PVA–DES–ASA nanofibers were prepared according to the following ratio, respectively: PVA (10.5%, w/v) dissolved in ultrapurified water; PVA (9%, w/v) and DES (5%, w/v) dissolved in ultrapurified water; PVA (9%, w/v), DES (5%, w/v) and ASA (5%, w/v) dissolved in ultrapurified water (Scheme 1). All the mixtures were stirred overnight at 80 °C to obtain electrospinning solutions according to reported procedures.^4,5,15^ For PVA–DES–honey nanofibers, PVA (8%, w/v) and DES (5%, w/v) were dissolved in ultrapurified water, and the mixture was stirred at 80 °C to obtain a homogenous and clear solution. Honey (5%, w/v) was then added to the PVA–DES solution and stirred at room temperature for 12 h to obtain a uniform and transparent solution according to reported procedures.^15,21^

#### 2.2.3 Fabrication of PVA–DES–honey/ASA nanofibers by electrospinning

PVA–DES–honey/ASA nanofibers were prepared by electrospinning according to reported procedures.^21–23^ An electrospinning solution of PVA (10.5%, w/v), an electrospinning solution of PVA (9%, w/v) and DES (5%, w/v), an electrospinning solution of PVA (9%, w/v), DES (5%, w/v) and ASA (5%, w/v), and an electrospinning solution of PVA (8%, w/v), DES (5%, w/v) and honey (5%, w/v) were sprayed from a 10 mL plastic syringe at a rate of 0.75 mL h^−1^. A voltage of 23 kV is applied to the needle, where the positive high voltage was 18 kV and the negative high voltage was 5 kV. The needle tip was 13 cm away from the collector. The electrospun nanofibers were removed the aluminum foil and dried under vacuum at room temperature overnight. The PVA, PVA–DES, PVA–DES–honey, PVA–DES–ASA nanofibers were further dried in a fume hood to remove residual solvent.

#### 2.2.4 Nanofiber characterization

Surface chemistry of the PVA, PVA–DES, PVA–DES–honey, PVA–DES–ASA nanofibers were characterized using a Nicolet 5700 spectrometer.^8,24^ Characterization of the elemental composition and molecular structure of nanofibers was conducted using the Axis Ultra X-ray photoelectron spectrometer.^9,25^ The morphology of the PVA, PVA–DES, PVA–DES–honey, PVA–DES–ASA nanofibers was observed using field emission scanning electron microscopy (FE-SEM S-4800, Hitachi Ltd, Japan). The obtained SEM micrographs were further analyzed using Image-Pro-Plus 6.0 (Media Cybernetics, Inc, USA) software to measure the fiber diameter. 150 fiber diameters were measured randomly on the SEM micrograph of each sample, according to the reported procedures.^8,24^

#### 2.2.5 Tensile properties

Mechanical properties of the PVA, PVA–DES, PVA–DES–honey, PVA–DES–ASA nanofibers were investigated according to reported procedures,^22,26^ using a universal tensile testing machine (DDL10, EDC-controller, Germany and China) with a crosshead rate of 5 mm min^−1^. The sample was stabilized on a frame with a gauge length of 10 mm, and equipped with a 100 N load cell. The final value was the average data of five specimens.

#### 2.2.6 Solubility test

For the dissolution test was conducted according to reported procedures.^14,27^ PVA, PVA–DES, PVA–DES–ASA and PVA–DES–honey nanofibers were put into glass vials. Photographic images were taken before and after dissolution. To record the process of dissolution, PVA–DES–ASA and PVA–DES–honey nanofibers were placed in Petri dishes, and videos were recorded while adding 1000 μL of ultrapurified water to the Petri dishes.

The disintegration of the nanofiber membrane in saliva was observed in artificial saliva solutions, according to reported procedures.^3,27^ It was produced by dissolving 1.9 mg KH_2_PO_4_, 80 mg NaCl, and 23.8 mg Na_2_HPO_4_ in 10 mL of ultrapurified water, followed by adjusting pH to 6.8 using phosphoric acid. A filter paper of appropriate size was placed in a 90 mm plastic Petri dish, and was thoroughly wetted with 10 mL of artificial saliva. Afterwards, the excess saliva was completely removed from the Petri dish, PVA–DES–ASA and PVA–DES–honey nanofibers were placed at the center of the filter paper. The disintegration process of PVA–DES–ASA and PVA–DES–honey nanofibers were recorded using a video camera. The presence of aspirin in PVA–DES–ASA nanofiber was analyzed by the Ultraviolet-Visible (UV-Vis) spectroscopy. First, 20 mg of PVA–DES–ASA nanofiber membrane was dissolved in 50 mL ultrapurified water, and then put 2 mL of the solution in a cuvette for UV-vis spectrophotometric analysis. A control group of 20 mg PVA nanofiber membrane was also dissolved in ultrapurified water for UV-vis spectrophotometer analysis.^5,28^

### 2.3 Antibacterial activity test

#### 2.3.1 Preparation of bacterial culture

*Escherichia coli* HB101 (*E. coli*) and *Staphylococcus aureus* ATCC 25923(*S. aureus*) were used as the model strains to test the antimicrobial activity of PVA–DES–honey nanofiber membranes. The two kinds of bacteria were separately streaked on the 20 mL solid lysogeny broth (LB) agar plates (0.2 g Bacto™ tryptone, 0.2 g sodium chloride, 0.1 g yeast extract and 0.24–0.3 g agar, pH 7), then placed in a 37 °C incubator for 15 h to culture single colony, according to reported procedures.^29,30^ The instruments and reagents were all sterilized, and all steps were carried out in a biological safety cabinet.

#### 2.3.2 Colony plate counts method

The antimicrobial properties of the nanofibers against *E. coli* and *S. aureus* was evaluated according to reported procedures.^31–33^ The PVA, PVA–DES and PVA–DES–honey nanofibers were sterilized under UV irradiation for 30 min separately, and then placed in 20 mL LB medium inoculated with a single colony of *E. coli* and *S. aureus*, respectively. The LB medium inoculated with a single colony of *E. coli* and *S. aureus* without nanofiber was considered as the control. The sample was then incubated with shaking at 200 rpm and 37 °C for 3 h. An aliquot of 100 μL bacterial solution was pipetted on the agar plate and spread it evenly and incubated at 37 °C for 12 h. The growth of colonies on the agar plates was recorded to evaluate the antimicrobial activity of PVA–DES–honey nanofibers.

#### 2.3.3 Quantitative analysis of antibacterial efficacy

The antibacterial activities of PVA–DES–honey nanofibers were further analyzed using a spectrophotometric method according to a reported procedure.^25^ Optical density measurements were taken at 600 nm (OD_600_ nm) of bacterial cell suspensions untreated blank and treated with PVA nanofibers, PVA–DES nanofibers and PVA–DES–honey nanofibers to quantify bacterial cells density and to evaluate the antimicrobial activity of PVA–DES–honey nanofiber membranes. Three nanofiber membranes of the same size were irradiated under ultraviolet light for 30 min for sterilization. The nanofiber membranes were then immersed in flask containing LB inoculated with *E. coli* and *S. aureus* cultures using a single colony, respectively. The samples were placed in a shaker at 37 °C for 200 rpm, and the bacterial cell density (OD_600_ nm) was measured every 2 to 6 h to monitor the antimicrobial activity of PVA–DES–honey nanofibers. The reduction of bacteria was calculated using the following eqn (1):

1 Bacterial reduction (%) = 100(*B* − *A*)/(*B*)

where *A* was the OD_600_ value of bacterial culture medium inoculated with the PVA–DES nanofibers and PVA–DES–honey nanofibers and blank, and *B* was the OD_600_ value of bacterial culture medium with the PVA nanofibers.^9,23^ The antimicrobial experiment was conducted in triplets.

### 2.4 Cell viability assay

NIH/3T3 and HepG2 cells as model cells to evaluate the cytocompatibility of PVA, PVA–DES, PVA–DES–ASA and PVA–DES–honey nanofibers. The nanofiber membranes were cut to the same size as a 24-well plate. All nanofiber membranes of the same size were irradiated with ultraviolet rays for 30 min, and were then the sterilized nanofiber membranes were placed in a 24-well plate. NIH/3T3 and HepG2 cells were cultured in a 10% fetal bovine serum and 90% DMEM (100 U mL^−1^ penicillin and 100 μg mL^−1^ streptomycin) solution, and were placed in a 37 °C, 5% CO_2_ incubator. A 0.25% trypsin solution (Invitrogen) was used to digest cultures every 3 days to keep the cells in exponential growth phase. To determine the cell viability on the nanofibers, cells (NIH/3T3 and HepG2) were digested with 0.25% trypsin (Invitrogen) and were seeded on the nanofibers in 24-well plates at a density of 3 × 10^4^ cells in 1 mL of cell culture medium and then incubated in an incubator at 37 °C for 24 h. After a predetermined incubation time, an aliquot of 300 μL fluorescein diacetate (FDA) solution was added to each well (dissolve FDA in acetone solution in advance to prepare a storage solution with a concentration of 1 mg mL^−1^, store in a refrigerator at −20 °C in the dark, dilute 100 times with PBS before use), and incubated in a 37 °C incubator for 5 min. The cells were then rinsed with PBS, imaged through an optical microscope to observe the cell morphology.^25,29,30^ The cell viability analysis was conducted in triplets. The instruments and reagents were all sterilized, and all steps were carried out in a biological safety cabinet.

### 2.5 *In vivo* wound healing studies

In this study, all procedures involving animal use have been authorized by the Animal Care and Use Committee of Northwest A&F University. To evaluate the wound healing ability of the PVA–DES–honey nanofiber membrane, healthy rat (Sprague-Dawley rat, female, 6 weeks) were randomly divided into 3 groups and with 5 rats in each group. After all rats were anesthetized with chloral hydrate (0.3 mg kg^−1^ body weight) in the abdomen, the dorsal area was shaved. Then a sterile scalpel was used to cut a round, full-thickness skin wound with a diameter of 8 mm on the back of each rat. The PVA, PVA–DES and PVA–DES–honey nanofiber membranes were all sterilized by ultraviolet light for 30 min, and then each of them was placed on the wound site for *in vivo* wound healing studies.^34,35^ PVA–DES–honey nanofibers were used as the positive control, while PVA and PVA–DES nanofibers were used as the negative control. Because the prepared nanofiber membranes have good water solubility, and the materials used for wound healing are all good natural antibacterial and anti-inflammatory materials, and they have good biocompatibility and good wound healing ability. Therefore, they can be directly adhered to and absorbed by the wound. Then, 15 rats were individually placed in cages for observation, and equal food and water were provided at a constant temperature. The wound area was imaged on the 0th, 3rd, 6th, 9th, and 12th days after surgery, and pictures of the wound area were taken to record the optical images of the wound. The wound area was accurately measured with professional software (Image-Pro-Plus 6.0). The wound area ratio is calculated using the following eqn (2):

2 Wound area ratio (%) = [*W*_(6,12)_/*W*_(0)_] × 100%

where *W*_(0)_ and *W*_(6,12)_ represent the exposed areas of the wounds on day 0, 6 and 12, respectively.^34,36,37^

## 3. Results and discussion

### 3.1 Synthesis and characterization of PVA–DES–honey/ASA nanofibers

The surface chemistry of the functional PVA–DES nanofibers were characterized by FT-IR analysis.

**Fig. 1:**
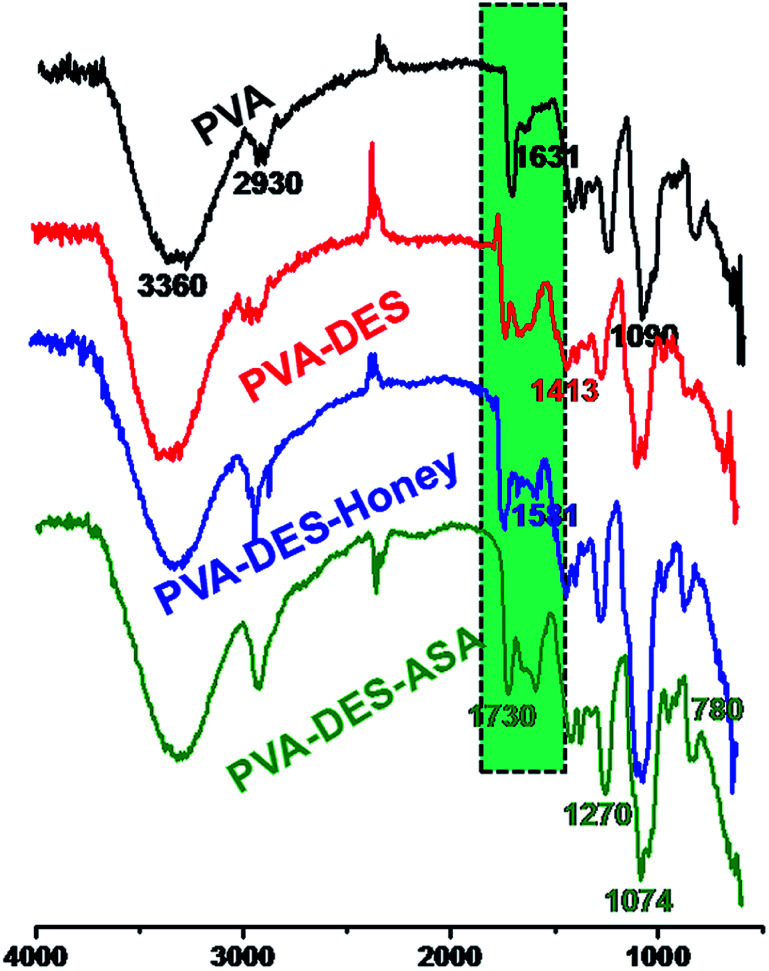
FT-IR spectra of PVA (black line), PVA–DES (red line), PVA–DES–honey (blue line) and PVA–DES–ASA nanofibers (green line).

Pure PVA spectrum (Fig. 1, black line) showed a main O–H stretching vibration peak at 3360 cm^−1^, C–H stretching vibration at 2930 cm^−1^, –C–C– stretching vibration peak at 1631 cm^−1^, C–O stretching vibration at 1090 cm^−1^, in agreement with Tang's report.^21^ FT-IR spectrum of PVA–DES nanofibers (Fig. 1, red line) had a peak at 1413 cm^−1^, which corresponded to C–N stretching of the choline chloride of DES. FT-IR spectrum of PVA–DES–honey (Fig. 1, blue line) had a peak at 1581 cm^−1^, which corresponded to N–H bending in the honey.^16,21,38^ FT-IR spectrum of PVA–DES–ASA nanofibers (Fig. 1, green line) had a peak at 1730 cm^−1^, indicative of C=O vibration, and peaks at 1270, 1074, and 780 cm^−1^, characteristic for benzene absorption.^11,28^ Comparison of the FT-IR spectra of the four nanofibers revealed a difference in the 1660–2000 cm^−1^, which was caused by the C–H bending vibration of the benzene ring and C–N stretching of the choline chloride. The results suggested that we successfully prepared the functional PVA–DES nanofibers (PVA–DES–honey, PVA–DES–ASA) with incorporated honey and ASA.

**Fig. 2:**
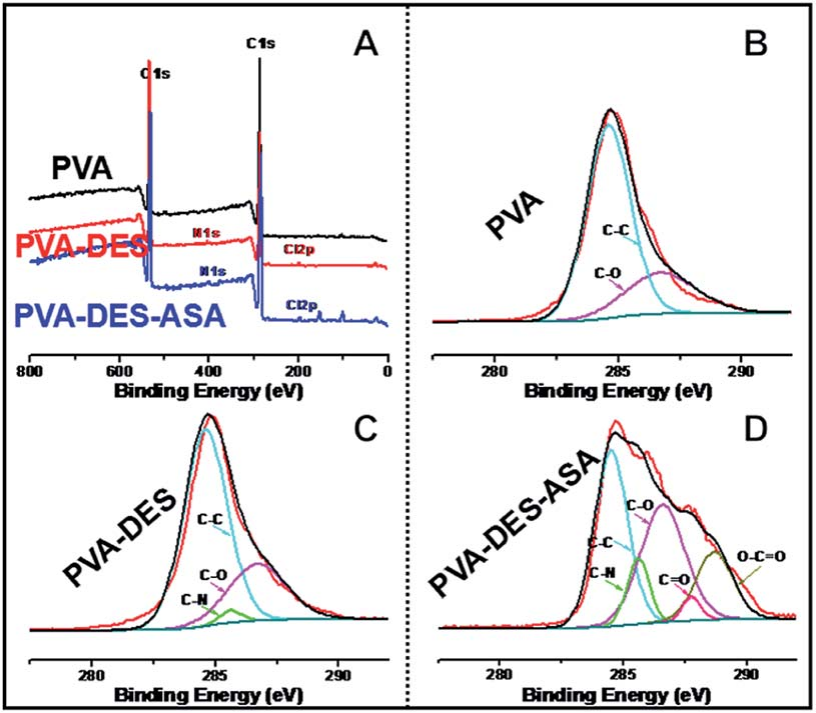
(A) XPS wide-scan spectra of PVA nanofibers (black line), PVA–DES nanofibers (red line) and PVA–DES–ASA nanofibers (blue line); (B) high-resolution XPS C 1s spectra of PVA nanofibers; (C) high-resolution XPS C 1s spectra of PVA–DES nanofibers; (D) high-resolution XPS C 1s spectra of PVA–DES–ASA nanofibers.

The surface chemistry of the functional PVA–DES nanofiber membranes were further analyzed using XPS analysis (Fig. 2). The wide-scan spectrum of PVA nanofibers showed only carbon (C 1s at 284.5 eV) and oxygen (O 1s at 531.0 eV) peaks (Fig. 2A). When DES was used, nitrogen (N 1s at 400.0 eV) and chlorine (Cl 2p at 197.0 eV) appeared in the XPS spectra of PVA–DES and PVA–DES–ASA nanofibers (Fig. 2A). The results suggested the introduction of DES changed the material chemistry of PVA. High-resolution scan of these nanofibers was conducted on the C 1s region (Fig. 2B–D). The control PVA nanofibers had peaks at 284.6 eV (C–C) and 286.7 eV (C–O) (Fig. 2B). The XPS spectra of PVA–DES nanofibers had a new bond at 285.6 eV (C–N) (Fig. 2C), indicative of the DES in nanofibers.^9,39^ After the introduction of ASA, the spectrum of PVA–DES–ASA showed Fig. 2D new peaks of C=O bond at 287.8 eV and O–C=O bond at 288.7 eV (Fig. 2D), indicative of successful incorporation of ASA into the nanofibers. It is worthwhile to note that the XPS of PVA–DES–honey was not analyzed due to the chemical complexity of honey. Overall, the above results again demonstrated the successful preparation of the functional PVA–DES nanofibers.

**Fig. 3:**
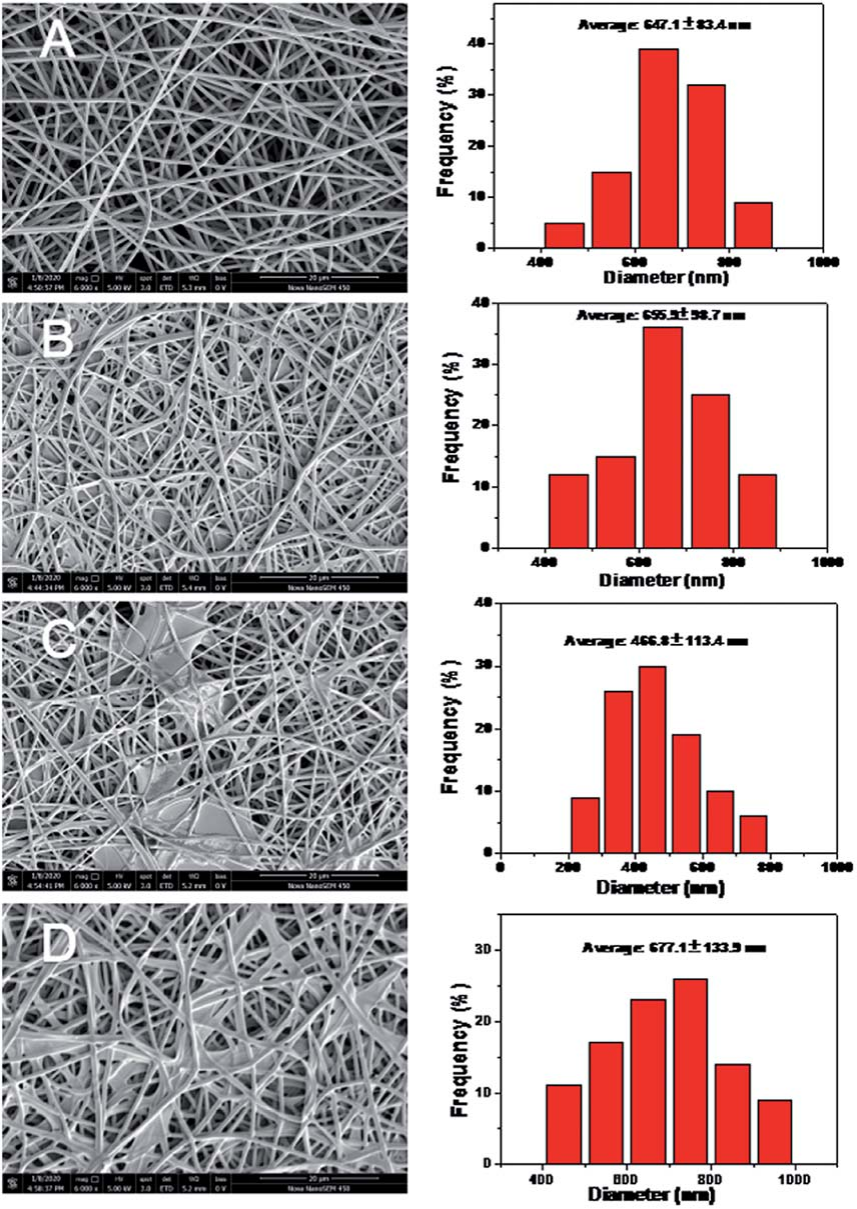
FE-SEM images and diameter distribution of different nanofiber membranes: (A) PVA, (B) PVA–DES, (C) PVA–DES–honey; (D) PVA–DES–ASA nanofibers.

The FE-SEM images of pure PVA, PVA–DES, PVA–DES–honey and PVA–DES–ASA nanofibers were shown in Fig. 3. Pure PVA nanofibers had a cylindrical shape, with uniform diameter and smooth surface, and the fibers had an intact morphology (Fig. 3A left).^40^ The average diameter of PVA nanofibers was 647.1 ± 83.4 nm, determined by software Image-Pro-Plus 6.0 (Fig. 3A right). Compared with PVA nanofibers, PVA–DES nanofibers are flattened (Fig. 3B left), with an average diameter of 655.9 ± 98.7 nm (Fig. 3B right). The morphology and the diameter distributions of the PVA–DES–honey nanofibrous were shown in Fig. 3C. When 5% honey was introduced, PVA–DES–honey nanofibers had a lower fiber diameter of 466.8 ± 113.4 nm comparing to PVA and PVA–DES nanofibers (Fig. 3C right). The change in fiber diameter was likely caused by a change in the conductivity of the solution. The conductivity of honey is closely related to its concentration.^21,40^ The higher conductivity of the solution, the more charge it carried in the jet, causing an increase in the elongation force, and a decrease in fiber diameters. Compared with PVA, PVA–DES nanofibers and PVA–DES–honey, PVA–DES–ASA nanofibers had the highest fiber diameter of 677.1 ± 133.9 nm (Fig. 3D right).

**Fig. 4:**
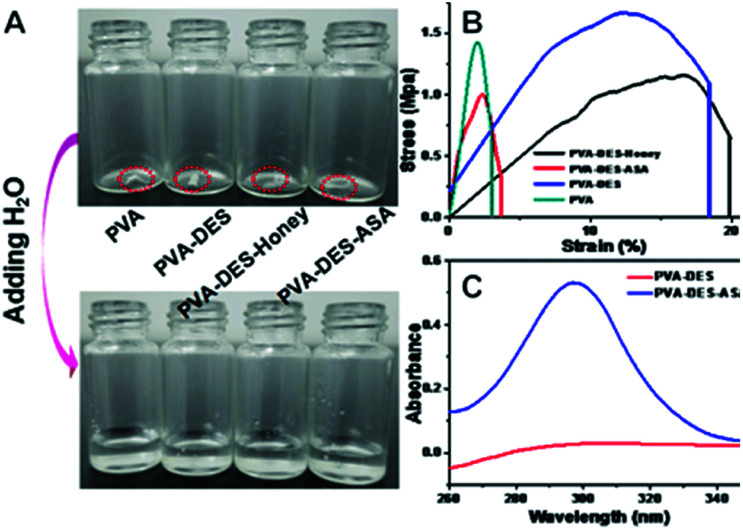
(A) Presentation of solubility behavior of PVA, PVA–DES, PVA–DES–honey and PVA–DES–ASA nanofibers. The photos were taken before nanofibers were in contact with water and after nanofibers were in contact with water. (B) Stress–strain curves of the nanofiber membranes of PVA, PVA–DES, PVA–DES–honey and PVA–DES–ASA nanofibers. (C) UV/vis spectra of PVA–DES nanofibers and PVA–DES–ASA nanofibers.

The ultraviolet-visible (UV-Vis) spectroscopy of the nanofibers in water solution were analyzed (Fig. 4C). ASA has a relatively poor solubility, which can typically be improved by embedding ASA in the PVA–DES (PVA–DES–ASA) nanofiber membrane, to improve local and low-dose applications and to achieve uniform distribution and rapid dissolution of ASA.^41^ As shown in Fig. 4C, the maximum absorption of PVA–DES–ASA solution was at 295 nm, suggesting the presence of ASA in the solution.^5^ In contrast, the control PVA–DES nanofibers did not show ultraviolet absorption. The results showed that ASA was successfully embedded in PVA–DES (PVA–DES–ASA) nanofiber membranes and the solubility of ASA was improved. The results suggested that the PVA–DES nanofiber film had the potential to improve the solubility of the encapsulated drugs for fast-dissolving drug delivery application.

When PVA–DES–honey and PVA–DES–ASA are utilized in the fast-dissolving drug delivery systems, the rapid dissolution or disintegration of the nanofiber membrane in the oral cavity can improve the effective bioavailability and therapeutic potential of the drugs. The dissolution characteristics of nanofibers were evaluated by adding water to the nanofibers (Fig. 4A and Video S1†). Once the PVA–DES–honey and PVA–DES–ASA nanofibers contacted with water, they immediately absorbed water and dissolved completely. A filter paper soaked with artificial saliva was used to imitate the humid environment in the oral cavity, and the disintegration of PVA–DES–honey and PVA–DES–ASA nanofibers in the oral cavity was further studied. As shown in Video S2,† PVA–DES–honey and PVA–DES–ASA nanofibers were instantly absorbed the artificial saliva, and then immediately dissolved or dispersed in saliva. The rapid dissolution of PVA–DES–honey and PVA–DES–ASA nanofibers suggested the following two points: first, the excellent hydrophilicity, wettability and water solubility of PVA can lead to the ability to dissolve rapidly; secondly, the high surface area and abundant porosity of the nanofibers allows the artificial saliva to quickly penetrate and quickly disintegrate the nanofiber films and to release encapsulated active ingredients.^27^ Flavoring agents were also incorporated into fast-dissolving drug delivery systems to reduce the difficulty of taking medication due to bitterness.^2,5,27^ Our results suggested our PVA-DES nanofiber-based fast-dissolving drug delivery systems have the potential for rapid and target drug delivery in applications such as oral ulcer stickers.

### 3.2 Antibacterial activity of PVA–DES–honey nanofibers

**Fig. 5:**
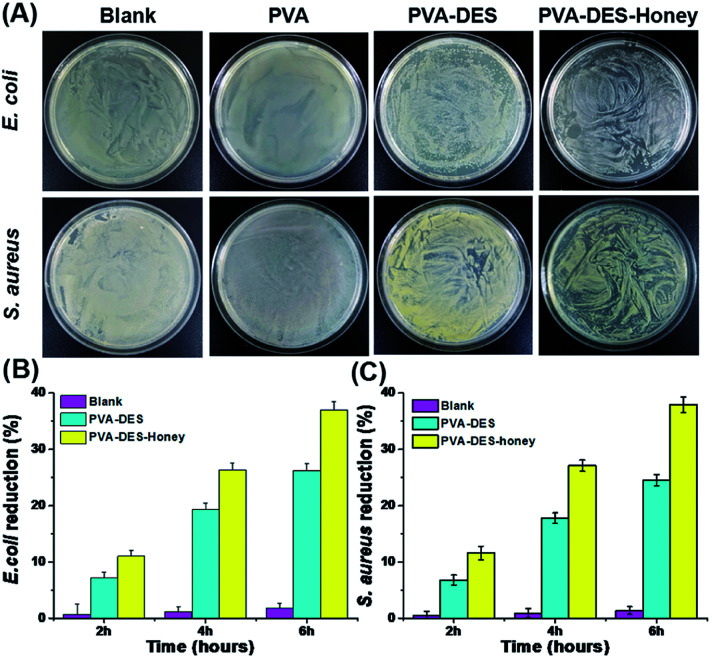
(A) Viable bacteria remaining in the LB-agar plates of *E. coli* and *S. aureus* after being treated with PVA, PVA–DES and PVA–DES–honey nanofibers and untreated blank control. (B and C) Percentage of bacterial reduction *vs.* time plots for *E. coli* (B) and *S. aureus* (C) culture medium inoculated with PVA, PVA–DES and PVA–DES–honey nanofibers and untreated blank control. Data are expressed as mean ± SD (*n* = 3).

*E. coli* and *S. aureus* were used to evaluate the antimicrobial activity of PVA–DES–honey nanofiber membranes through the classic colony counting method, with untreated blank, PVA and PVA–DES as controls. As shown in Fig. 5A, regardless of *E. coli* and *S. aureus*, a large number of bacterial colonies can be observed on the agar for bacteria cultured with the control PVA nanofiber membranes and untreated blank, suggesting the pure PVA nanofiber membrane could not inhibit the growth of bacteria. In contrast, bacteria cultured with PVA–DES and PVA–DES–honey nanofiber membranes have only a few bacterial colonies on agar medium. In particular, the PVA–DES–honey nanofiber membrane has the smallest number of bacteria growth, suggesting the nanofiber membrane has the strongest antibacterial effect. The results were in agreement with previous reports that honey has good antibacterial properties.^21,25^

We also evaluated the antibacterial activity of PVA–DES–honey nanofiber membrane by measuring the optical density at 600 nm (OD_600_ nm) of bacterial cell suspensions treated PVA–DES–honey nanofibers, with untreated blank, PVA and PVA–DES treatment as controls, to quantitatively evaluate the antibacterial effect of PVA–DES–honey nanofibers. As shown in Fig. 5B and C, the untreated *E. coli* and *S. aureus* are slightly less than those of the PVA nanofiber membranes after incubation, indicating that the PVA nanofiber membranes showed no antibacterial activity against *E. coli* and *S. aureus* after incubation, in fact there was a slightly accelerative growth of these two bacteria. Both PVA–DES and PVA–DES–honey showed bacterial reduction when incubated with both *E. coli* and *S. aureus*. As shown in Fig. 5B, the reduction of PVA–DES–honey nanofibers treated samples had *E. coli* reduction of 11.0%, 26.3%, and 37.0%, after 2 h, 4 h, and 6 h incubation time, respectively. The PVA–DES treated samples had lower *E. coli* reduction of 7.2%, 19.3%, and 26.2%, after 2 h, 4 h, and 6 h incubation time, respectively. Similarly, the PVA–DES–honey enhanced the *S. aureus* reduction from 6.8% to 11.6%, 17.8% to 27.1%, 24.5% to 37.9%, after 2 h, 4 h, 6 h of incubation time, respectively, comparing to the control PVA–DES treatment (Fig. 5C). Overall, the antibacterial efficiency of the PVA–DES–honey nanofiber membrane against both *E. coli* and *S. aureus* increased as the incubation time increased. The PVA–DES–honey had stronger antibacterial activities against both Gram-positive and Gram-negative strains than PVA–DES, and agreed with previous reports.^21,42^ The antibacterial ability of PVA–DES and PVA–DES–honey nanofiber membranes can be attributed to the following two points: firstly, the quaternary ammonium salt component of choline chloride in DES can result bacterial death by destroying the bacterial cell membrane;^19^ secondly, honey contains antibacterial substances (*e.g.* lysozyme and glucose oxidase), which hinder the growth of bacteria to due low pH and high osmotic pressure.^21,40,42^ The results showed that PVA–DES–honey nanofiber membrane possess excellent antimicrobial activity.

### 3.3 Assessment of the cell viability

**Fig. 6:**
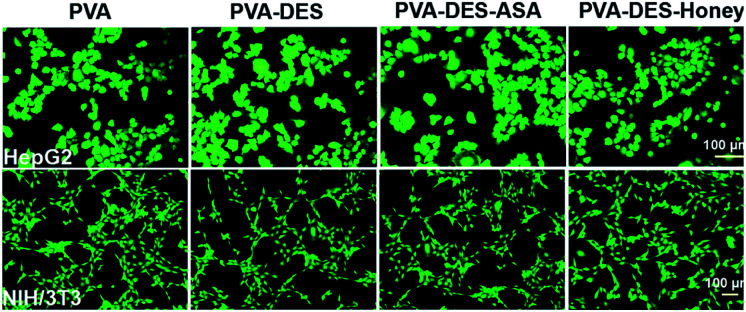
Fluorescent micrographs of 3 × 10^4^ cells per mL NIH/3T3 and HepG2 cells cultured on PVA, PVA–DES, PVA–DES–ASA and PVA–DES–honey nanofibers for 24 h at 37 °C.

**Fig. 7:**
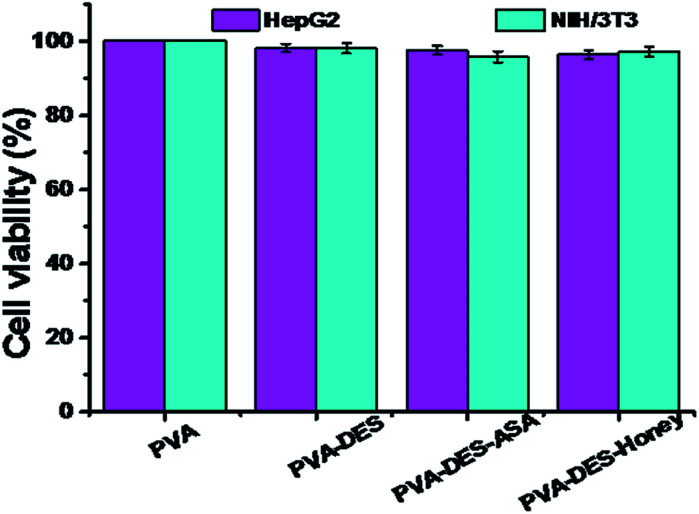
Quantitative analysis of the cell viability after incubation on PVA, PVA–DES, PVA–DES–ASA and PVA–DES–honey nanofibers for 24 h at 37 °C, which correspond to the micrographs in Fig. 6. Data are expressed as mean ± SD (*n* = 3).

The cell biocompatibility of the functional PVA–DES nanofibers was studied (Fig. 6). After NIH/3T3 and HepG2 cells were cultured on four nanofiber membranes of PVA (control), PVA–DES (control), PVA–DES–ASA and PVA–DES–honey nanofibers for 24 h, FDA dyes were used to stain cells to further determine cell viability.^8,25^Fig. 6 showed the fluorescent microscopic images of NIH/3T3 and HepG2 cells were cultured on four different nanofibers. The cells cultured on all nanofiber samples showed a flattened fusion-like layer on the surface, and exhibited a good growth state. Image-Pro-Plus 6.0 was used to count the cells for further quantify the cell viability (Fig. 7).

The cells incubated on all the nanofiber samples had more than 90% of cell viability. The results agreed with previous reports, where choline chloride, mannose, ASA and honey have good cytocompatibility,^18,21^ The results suggested that the presence of DES–ASA and DES–honey was not only non-toxic to cells, but is beneficial to the adhesion and proliferation of NIH/3T3 and HepG2 cells. Overall, our research illustrated that the functional PVA–DES nanofibers exhibited excellent cytocompatibility.

### 3.4 Assessment of the wound healing ability

**Fig. 8:**
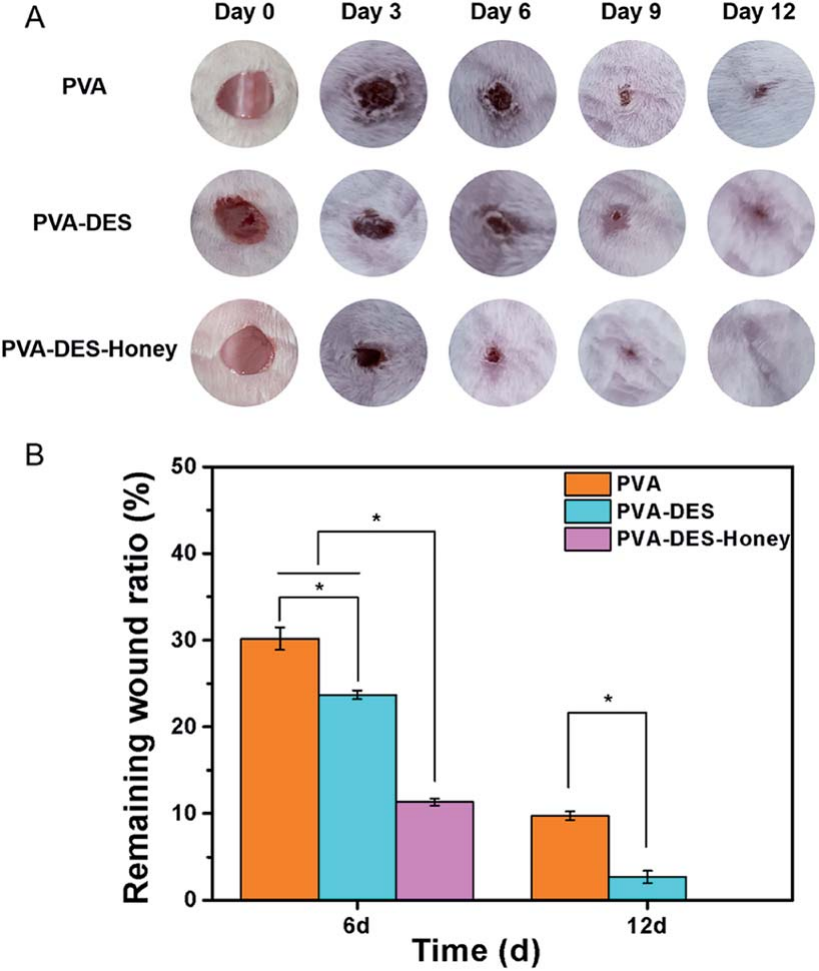
(A) Photographs of the wound treated with PVA, PVA–DES and PVA–DES–honey nanofibers at days 0, 3, 6, 9, and 12, respectively. (B) Wound area ratio of the wound treated with PVA, PVA–DES and PVA–DES–honey nanofibers after days 6 and 12, respectively, which correspond to the micrographs in (A). Data are expressed as mean ± SD (*n* = 5).

Rat was used as a model to evaluate the wound healing ability of PVA–DES–honey nanofiber membrane, with PVA and PVA–DES nanofiber membranes as controls. The wound area was imaged at 0, 3, 6, 9, and 12 days after the surgery to record the changes in wound size over time (Fig. 8A). Image-Pro Plus 6.0 software was used to measure the wound area, and the wound area ratio is calculated by the wound area at each time point over the original wound area (Fig. 8B).^43,44^ After day 6, the wound area after treatment with pure PVA nanofiber membrane was reduced to 31.8%. The wound area of PVA–DES and PVA–DES–honey nanofiber membranes decreased from 100% to 23.7% and 14.8%, respectively. Compared with PVA nanofiber membranes, both PVA–DES nanofiber membranes and PVA–DES–honey nanofiber membranes reduced wounds, while PVA–DES–honey nanofiber membranes showed higher wound closure rates. After 9 days of treatment, the wound in the PVA–DES–honey nanofiber membrane treatment group was almost completely closed. After 12 days, the wound after treatment with PVA–DES–honey nanofiber membrane was completely healed. The wounds treated with pure PVA and PVA–DES nanofiber membranes were almost completely closed after 12 days. Overall, the PVA–DES and PVA–DES–honey nanofibers significantly increased the wound closure rate.

There are several explanations for the excellent role of PVA–DES–honey nanofibers in promoting wound healing. Firstly, owing to the hydrophilicity and excellent water solubility of PVA and honey, the PVA–DES–honey nanofiber membrane can easily adhere to the wound and release drugs, and keep the wound in an ideal moisture state.^21,42^ Secondly, the quaternary ammonium salt of choline chloride has good antibacterial and anti-inflammatory effects, and mannose can heal wounds and resist inflammation. The combined effect of the two can inhibit the growth and proliferation of most pathogenic bacteria, and can greatly reduce the risk of wound infection. At the same time, both choline chloride and mannose are good nutrients. Finally, honey has good antibacterial, anti-inflammatory, anti-oxidant and antibiotic properties, which can prevent wound infection, inhibit inflammation, enhance the tissue repair process, and support regenerative healing. In addition, honey can also provide a moist healing environment on the wound and accelerate healing. It also can maintain acidic pH, which is conducive to exudation and debridement, has a good effect of promoting wound healing.^21,40,42^ All of these factors are conducive to PVA–DES–honey nanofiber membrane become an ideal wound dressings.

## 4. Conclusion

In this study, we successfully prepared fast-dissolving drug delivery systems by electrospinning for the simultaneous release of choline chloride, mannose, honey, and acetylsalicylic acid. The PVA–DES based fast-dissolving drug delivery systems can be potentially used as oral instant film, oral ulcer stickers, and wound dressings. The chemical composition, structure, and morphology were further characterized based on FTIR, XPS, and FE-SEM, which proved the successful preparation of PVA–DES–honey and PVA–DES–ASA nanofibers. Compared with PVA and PVA–DES nanofibers, PVA–DES–honey nanofibers showed significant antimicrobial activity, while showing good cytocompatibility and the ability to heal wounds. Therefore, PVA–DES–honey nanofibers are expected to be used in wound dressing applications. Finally, the rapid dissolution and aspirin encapsulation tests of PVA–DES–ASA successfully demonstrated its potential in oral mucosal release systems and rapid dissolution oral drug delivery systems. Overall, our work opens up new opportunities for designing cheaper and biodegradable delivery mechanisms in the pharmaceutical field, using simple and universal technologies.

## Conflicts of interest

There are no conflicts to declare.

## Supplementary Material

RA-011-D0RA08755F-s001

RA-011-D0RA08755F-s002
